# Fucosylated exosomal miRNAs as promising biomarkers for the diagnosis of early lung adenocarcinoma

**DOI:** 10.3389/fonc.2022.935184

**Published:** 2022-08-12

**Authors:** Xiongfeng Chen, Lili Yu, Kun Hao, Xiaoqing Yin, Mingshu Tu, Liqing Cai, Liangming Zhang, Xiaojie Pan, Qi Gao, Yi Huang

**Affiliations:** ^1^ Shengli Clinical Medical College, Fujian Medical University, Fuzhou, China; ^2^ Department of Scientific Research, Fujian Provincial Hospital, Fuzhou, China; ^3^ Department of Clinical Laboratory, Fujian Provincial Hospital, Fuzhou, China; ^4^ Research and Development Center, Beijing Glyexo Gene Technology Co., Ltd, Beijing, China; ^5^ Integrated Chinese and Western Medicine College, Fujian University of Traditional Chinese Medicine, Fuzhou, China; ^6^ Department of Thoracic Surgery, Fujian Provincial Hospital, Fuzhou, China; ^7^ Central laboratory, Fujian Provincial Hospital, Fuzhou, China; ^8^ Center for Experimental Research in Clinical Medicine, Fujian Provincial Hospital, Fuzhou, China

**Keywords:** lung adenocarcinoma, fucose-captured strategy, serum exosomal miRNA, biomarker, early LUAD diagnosis

## Abstract

**Background:**

Considering the absence of apparent symptoms at the early stage, most patients with lung adenocarcinoma (LUAD) present at an advanced stage, leading to a dismal 5-year survival rate of <20%. Thus, finding perspective non-invasive biomarkers for early LUAD is very essential.

**Methods:**

We developed a fucose-captured strategy based on lentil lectin-magnetic beads to isolate fucosylated exosomes from serum. Then, a prospective study was conducted to define the diagnostic value of serum exosomal miRNAs for early LUAD. A total of 310 participants were enrolled, including 146 LUAD, 98 benign pulmonary nodules (BPNs), and 66 healthy controls (HCs). Firstly, exosome miRNAs in the discovery cohort (n = 24) were profiled by small RNA sequencing. Secondly, 12 differentially expressed miRNAs (DEmiRs) were selected for further screening in a screening cohort (n = 64) by qRT-PCR. Finally, four candidate miRNAs were selected for further validation in a validating cohort (n = 222).

**Results:**

This study demonstrated the feasibility of a fucose-captured strategy for the isolation of fucosylated exosomes from serum, evidenced with exosomal characteristics identified by transmission electron microscopy (TEM), nanoparticle tracking analysis (NTA), and Western blotting, as well as rapid and convenient operation of <10 min. Furthermore, a miRNA panel for early LUAD composed of miR4732-5p, miR451a, miR486-5p, and miR139-3p was defined with an AUC of 0.8554 at 91.07% sensitivity and 66.36% specificity.

**Conclusions:**

The fucose-captured strategy provides a reliable, as well as rapid and convenient, approach for the isolation of tumor-derived exosomes from serum. A four-fucosylated exosomal miRNA panel presents good performance for early LUAD diagnosis.

## Background

Lung cancer, an extremely invasive malignancy, has become the leading cause of cancer-related death. As a predominant histologic subtype of lung cancer, lung adenocarcinoma (LUAD) accounts for about 40% of all cases (1). Due to the absence of apparent symptoms at the early stage, most patients with LUAD present at an advanced stage, leading to a dismal 5-year survival rate of less than 20% (2). However, the 5-year survival rate of LUAD patients at stage I who underwent surgical resection has been demonstrated to reach as high as 92% (3, 4). Therefore, early diagnosis and timely treatment are of great significance for LUAD prognosis. As a non-invasive approach, serological biomarkers have the advantages of convenience and safety in the screening of symptomatic populations. Nevertheless, the current tumor markers, such as carcinoembryonic antigen (CEA), cytokeratin 19 fragment (CYFRA 21–1), carbohydrate antigen 19-9 (CA19-9), and neuron-specific enolase (NSE), meet a different degree of detection sensitivity or specificity problems for LUAD diagnosis (5, 6). Therefore, finding new prospective non-invasive biomarkers is very essential for patients with LUAD.

Exosomes are 30–200nm membranous extracellular vesicles (EVs) in all body fluids (7) containing various functional biomolecules, such as protein, lipid, DNA, RNA, and miRNA (8). Recent studies have shown that cargoes in serum exosomes might serve as valuable serological biomarkers for early diagnosis of malignancies (9, 10). As the most important exosomal cargoes, miRNAs are small non-coding RNAs with 19–25 nucleotides in length and present the ability of regulating gene expression by binding to the 3′ untranslated regions of target mRNA (11). Interestingly, exosome-mediated miRNA transfer is revealed to be an effective strategy for miRNAs to exert their effects on tumor development (12). Some studies have shown that miRNA sorting is common during vesicle biogenesis, so specific miRNAs are enriched in exosomes (13). Moreover, exosomal miRNAs are more stable than extracellular miRNAs in the circulatory system because exosomes protect them from ribonuclease degradation (14). Therefore, exosomal miRNA detection has been recommended to be a promising non-invasive approach for malignancies at the early stage (15, 16).

Mounting lines of studies have shown the potentials of exosomal miRNAs for the diagnosis of lung cancer. For example, exosomal miR-17-5p was reported to be closely correlated with non-small cell lung cancer (NSCLC) and might serve as a diagnostic biomarker for patients with NSCLC (17). In addition, it was demonstrated that four LUAD-specific exosomal miRNAs (miR-181-5p, miR-30a-3p, miR-30e-3p, miR-361-5p) and three squamous cell carcinoma (SCC)-specific exosomal miRNAs (miR-10b-5p, miR-15b-5p, miR-320b) were valuable for early NSCLC diagnosis (18). A recent study by Zhang et al. (19) revealed that serum exosomal hsa-miR-20b-5p and hsa-miR-3187-5p are effective NSCLC biomarkers, manifested with the area under the receiver operating characteristic (ROC) curves (AUCs) of 0.810 and 0.673, respectively, and combination of hsa-miR-20b-5p and hsa-miR-3187-5p improved the AUC to 0.838. Nevertheless, exosomal miRNAs for early diagnosis of LUAD have to meet two main challenges at present. Firstly, it is difficult to enrich tumor-derived exosomes because current isolation methods such as ultracentrifugation (UC), superfiltration centrifugation, and Polyethylene Glycol (PEG) precipitation, to name a few, obtain the exosomes released from various cells more than they do from tumor cells (20). Secondly, most studies mainly consider the differences of exosomal miRNAs between lung cancer and healthy control (HC) but do not consider the differences of exosomal miRNAs between lung cancer and benign pulmonary nodule (BPN), so it is difficult to distinguish lung cancer from BPNs. Therefore, it is very essential to develop a new strategy to define valuable exosomal miRNAs for patients with LUAD at an early stage.

Previous studies have revealed that lectins can be used to isolate glycosylated extracellular vesicles (EVs). For example, lectin microarray technology was used to isolate glycosylated EVs from a diverse panel of human cell lines (T cells, melanoma, and colon cancer) and the physiological fluid breast milk (21). In addition, the STL lectin, binding the N-acetylglucosamine and lactosamine residues, also exhibited high affinity and specificity when isolating EVs from healthy urine samples (22). Recently, it has been reported that small EVs can be captured from melanoma, glioblastoma, and lung and colon cancer cells by coupling a high mannose-type glycan-specific lectin to beads (23). Herein, we first report that serum exosomes were isolated by a developed fucose-captured strategy based on lentil lectin (LCA)-magnetic beads. A prospective study was designed to collect and analyze serum samples from 310 subjects. Small RNA sequencing and qRT-PCR were performed to discover, screen, and verify new non-invasive biomarkers for early LUAD. The screening workflow for identifying exosomal miRNAs for diagnosing early LUAD is shown in **Figure S1**.

## Materials and methods

### Subjects and clinical samples

A total of 310 participants were enrolled in this study, including 146 patients with LUAD at stage I, 98 patients with BPNs, and 66 HCs. They were randomly assigned to discovery, screening, and validating cohorts (**Table 1**). LUAD patients were diagnosed by histological examination, and the tumor stage of LUAD was estimated according to TNM classification of the International Association for the Study of Lung Cancer (IASLC) eighth edition (24). The HC subjects received health examination in the physical examination center of Fujian Provincial Hospital and had no evidence of disease, including malignant tumor and BPN. This study was conducted in accordance with the International Ethical Guidelines for Biomedical Research Involving Human Subjects (CIOMS) and approved by the institutional review board (IRB) of Fujian Province hospital (ID: K2018-12-040). Participants signed informed consents before sample collection.

**Table 1:** Clinical characteristics of the discovery, screening, and validating cohorts.

		Discovery cohort (n = 24)			Screening cohort (n = 64)			Validating cohort (n = 222)		
Characteristics		LUAD (Stage I)	BPN	HC	LUAD (Stage I)	BPN	HC	LUAD (Stage I)	BPN	HC
Number		**8**	**8**	**8**	**26**	**26**	**12**	**112**	**64**	**46**
Age		51.00 ± 10.45	60.63 ± 12.96	51.13 ± 14.49	57.08 ± 9.76	53.15 ± 9.34	54.20 ± 10.95	59.80 ± 9.89	57.39 ± 10.13	54.91 ± 9.33
Gender	Men	3 (37.5%)	5 (62.5%)	6 (75.0%)	12 (46.2%)	15 (57.7%)	7 (58.3%)	55 (49.1%)	35 (54.7%)	25 (54.3%)
	Women	5 (62.5%)	3 (37.5%)	2 (25.0%)	14 (53.8%)	11 (42.3%)	5 (41.7%)	57 (50.9%)	29 (45.3%)	21 (45.7%)
Smoking history (%)		3 (37.5%)	4 (50%)	3 (37.5%)	11 (42.3%)	12 (46.2%)	5 (41.7%)	43 (38.4%)	29 (45.3%)	17 (36.9%)

LUAD, lung adenocarcinoma; BPN, benign pulmonary nodule; HC, healthy control; SD, standard deviation.

n in bold represents the total number of subjects included in the corresponding cohort.

In this study, 5 ml of peripheral blood from each subject was collected and the serum was separated at 3,000 rpm for 5 min and stored at −80°C until use.

### Isolation of serum exosomes by fucose-captured strategy

The serum exosomes were isolated by GlyExo-Capture according to a standard protocol (**Table S1**). In brief, the serum samples were incubated in the presence of LCA-immobilized beads, and the highly fucosylated exosomes can bind to LCA for separation. The specific steps are as follows: Firstly, 250 μl of serum sample was added to 2.0 ml EP tube. Secondly, 750 μl of LCA-coupled magnetic beads solution (MBL) was added, and it was incubated at room temperature for 1 min. Thirdly, it was placed on the magnetic frame to separate the magnetic beads and the supernatant, and the supernatant was discarded after standing for 1 min. Then, 600 μl of washing solution (WBL) was added, and the supernatant was discarded after placing it on the magnetic frame for 1 min. Finally, 250 μl of elution buffer (EBL) was added, and the supernatant was transferred to the new EP tube after incubating at room temperature for 1 min.

### Isolation of serum exosomes by ultracentrifugation

The isolation of serum exosomes *via* UC was performed as previously described (7). Briefly, the serum samples were centrifuged at 3,000 g for 15 min at 4°C to remove cell debris. Then, the collected supernatant was ultracentrifuged at 100,000 g for 1.5 h at 4°C (CP100NX; Hitachi). The exosome pellets were resuspended in phosphate-buffered saline (PBS) for further analysis.

### RNA isolation and analysis

Total RNA was extracted and purified using miRNeasy^®^ Mini kit (Qiagen, Cat. No. 217,004) according to standard protocol. RNA degradation and pollution, especially DNA contamination, were monitored on 1.5% agarose gel. RNA concentration, purity, and integrity were assessed using high-sensitivity RNA Cartridge of qsep100 automatic nucleic acid analysis system (Bioptic Inc., LA, USA).

### Library preparation and sequencing of small RNA

Sequencing libraries were constructed with 5 ng of RNA obtained from serum EVs using NEBNext1 Small RNA Library Prep Set for Illumina (Multiplex Compatible) (NEB, Cat. No. E7330) following manufacturer’s protocol. The sequencing library size selection was done using the E-Gel SizeSelect II gel of the E-Gel Power Snap electrophoresis system (Thermo Fisher Scientific Inc., MA, USA). Quality and concentration of cDNA libraries were checked, and then groups of 24 samples were pooled at the same concentration before sequencing in Illumina NextSeq 550 Sequencing System (75 nt, single read).

### Transmission electron microscopy

A total of 4 μl of suspended exosomes were dropped onto a copper mesh and left standing for 1 min, and the excess liquid was sucked from the edge of the copper mesh with filter paper. Then, the negative staining solution (0.5% uranium acetate aqueous solution, pH 4.5) was dropped for dyeing for 1 min, and the negative staining solution was sucked with filter paper. This step was repeated twice. Finally, exosomes were observed with transmission electron microscopy (TEM) on the FEI Tecnai Spirit 120KV.

### Nanoparticle tracking analysis

Exosomes isolated from serum were diluted 100- to 1,000-fold by a Nanosight NS 300 system (NanoSight Technology, Malvern, UK). Samples were manually injected into the sample chamber at ambient temperature. Each sample was configured with a 488-nm laser and a high-sensitivity scientific complementary metal oxide semiconductor camera and measured in triplicate under the camera setting. The acquisition time was set to 30 s, and the detection threshold was set to 7. At least 200 completed tracks were analyzed and obtained for each video. Finally, nanoparticle tracking data of exosomes were analyzed using the nanoparticle tracking analysis (NTA) analytical software (version 2.3).

### Western blotting

The protein concentration of the purified exosome solution was determined by a bicinchoninic acid (BCA) kit (Thermo Fisher Scientific, Waltham, MA, USA). The samples were mixed with an equal amount of loading buffer and then denatured in boiling water for 10 min. Sodium dodecyl sulfate-polyacrylamide gel electrophoresis (SDS-PAGE; 12.5%) was performed, and the samples were then transferred onto a polyvinylidene fluoride (PVDF) membrane. The membrane was blocked with 5% skimmed milk for 1 h and washed with Tris-buffered saline containing 0.2%–0.4% Tween-20 (TBST) three times. Exosome-panel antibodies (CD9, CD63, CD81, and calnexin; ab275018; Abcam, UK) were added, respectively, and the membrane was incubated at 4°C overnight. After washing with TBST three times, horseradish peroxidase-conjugated goat anti-rabbit IgG was added and incubated at room temperature in the dark for 1 h. Immunoreactive bands were developed by enhanced chemiluminescence reaction (Thermo Fisher, USA) following standard protocols, and a gel imager was used to take photographs.

### Small RNA sequencing data analysis

The adapter sequences were removed from raw single reads, and low-quality sequences (base quality <20) of the reads were further trimmed by Trim Galore. The trimmed readings were filtered by rRNA, tRNA, sRNA, and snRNA annotated in rfam and ensembl databases. The exon mRNA was then filtered because it is known that more than 70% of miRNA may exist in introns. The miRNAs were identified and quantified by using miRDeep2 v2.0.1.3 (25). Read counts of miRNAs were corrected to remove the effect of library size differences. The expression levels of miRNAs were quantified by reads per million (RPM) and then were converted to log_2_(RPM+1). A permutation test was performed to evaluate the differential expression of exosomal miRNAs between different groups, and *P* < 0.05 was considered a significant difference.

### Functional analysis of candidate miRNAs

Target genes of candidate miRNAs were predicted, and then Kyoto Encyclopedia of Genes and Genomes (KEGG) analysis was performed using OmicsBean Cancer data analysis tool (http://www.omicsbean-cancer.com/). Finally, miRNA–mRNA interaction network was constructed by using Cytoscape software.

### Expression levels of serum exosomal miRNAs by qRT-PCR

Strand cDNA was synthesized using miRcute plus miRNA cDNA first strand synthesis kit (Cat. No. KR211) according to standard protocol (Tiangen Biotech Co. Ltd., Beijing, China). The qRT-PCR of target miRNAs was performed by miRcute plus miRNA qPCR kit (SYBR Green) (Tiangen Biotech Co. Ltd., Beijing, China) on the ABI 7500 real-time PCR system (Applied Biosystems, CA, USA). The miRNA expression was quantified using 2^-ΔΔCt^ method and was normalized to miR20a. The specific primers used for qRT-PCR are listed in **Table S2**.

### Statistical analysis

Statistical analysis was performed using GraphPad Prism 8.0 and R 4.1.0 software. The Wilcoxon rank-sum test and the Mann–Whitney U-test were used to compare continuous variables. ROC curves and AUC were applied to evaluate candidate miRNAs’ diagnostic performance. The validated biomarkers were fitted to logistic regression model. Data were shown as mean ± SD, and *P* < 0.05 was considered statistically significant.

## Results

### Isolation and identification of serum-derived exosomes

**Figure 1:**
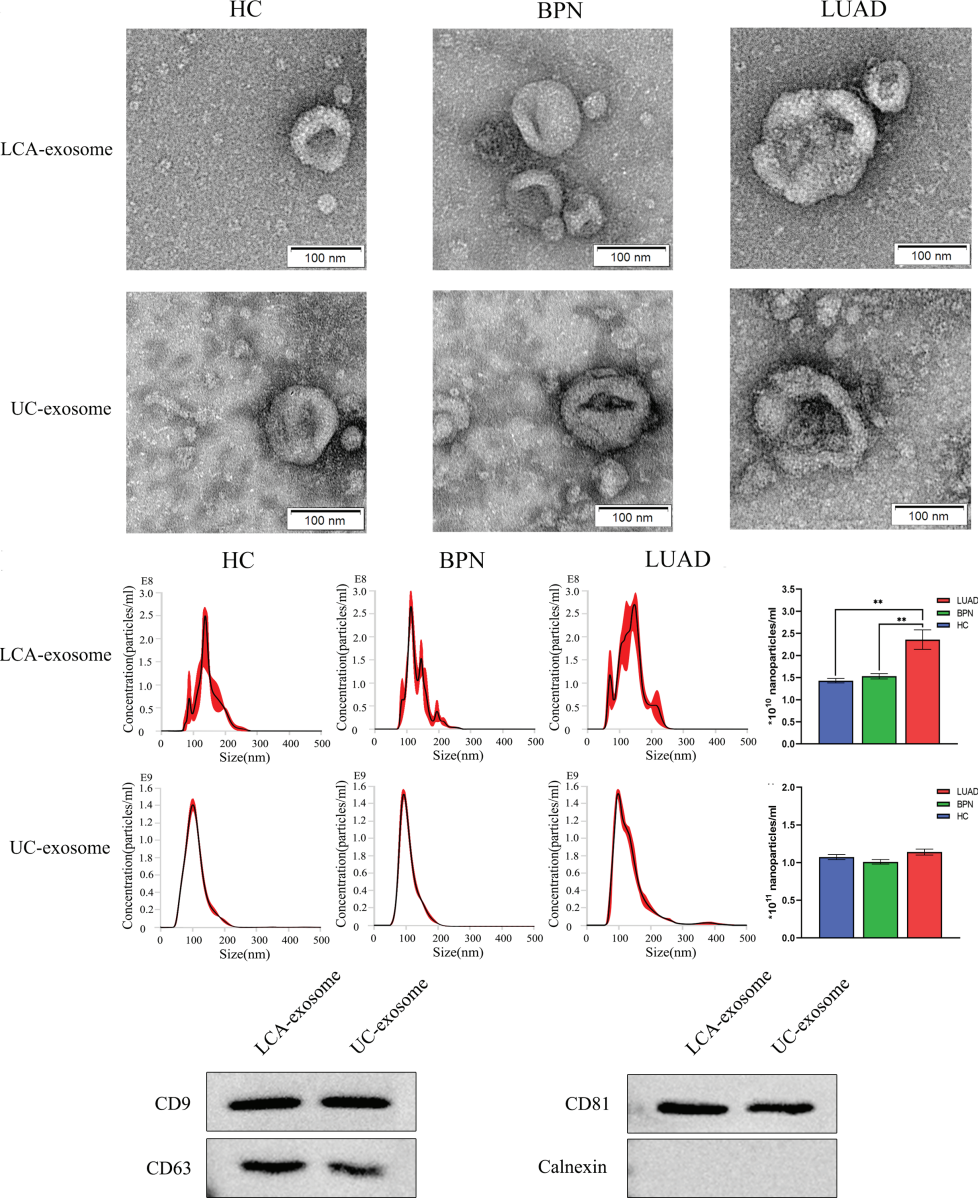
Identification of serum-derived exosomes isolated by LCA and UC methods. **(A)** Representative TEM images of exosomes isolated by LCA and UC methods; scale bars are 100 nm. **(B)** Representative graph of exosome concentration and size distribution as measured by NTA. Unpaired t-test was performed; **P < 0.01 vs. Other group. **(C)** Western blot images showing the expression levels of the exosomal proteins CD9, CD63, CD81, and calnexin. LUAD, lung adenocarcinoma; BPN, benign pulmonary nodule; HC, healthy control; LCA, lentil lectin; UC, ultracentrifugation; TEM, transmission electron microscopy; NTA, Nanoparticle tracking analysis.

To enrich tumor-derived exosomes in serum, we developed a Glyexo-capture strategy to isolate exosomes. This strategy used LCA-magnetic beads to couple fucose on the exosome membrane to separate exosomes from serum. It has been reported that abnormal activation of fucosyl transferase occurs in tumor patients and leads to the aberrant elevation of fucosylated glycoproteins on the surface of the tumor cell membrane (26, 27). The exosomes isolated from serum were characterized by TEM, NTA, and Western blotting. The isolated exosomes presented a typical membrane structure with a size of approximately 30–200 nm as observed by TEM (**Figure 1A**). Further measurements by NTA showed that an average particle size of exosomes separated by fucose-captured technique (HC: 147.3 ± 1.5 nm; BPN: 136.8 ± 2.0 nm; LUAD: 146.0 ± 8.8 nm; n = 3) was larger than that of exosomes separated by UC (HC: 108.4 ± 0.9 nm; BPN: 110.8 ± 0.3 nm; LUAD: 137.6 ± 7.76 nm; n = 3) but conformed to the particle size range of exosomes (**Figure 1B**). Moreover, the yield of serum exosomes isolated by Glyexo-capture was about 20% of that by UC (**Figure 1B**). Additionally, compared to the HC or BPN group, the concentration of nanoparticles from the LUAD group was markedly higher when the serum exosomes were isolated by Glyexo-capture (HC: 1.43E+10 ± 4.77E+08 particles/ml; BPN: 1.53E+10 ± 5.53E+08 particles/ml; LUAD: 2.36E+10 ± 2.21E+09 particles/ml; n = 3, *P* < 0.01) (**Figure 1B**). Finally, Western blot analysis showed that the released vesicles isolated by both fucose-captured technique and UC expressed the proteins including CD9, CD63, and CD81 enriched in exosomes and were absent with calnexin, a contamination marker from the endoplasmic reticulum (**Figure 1C**). These results indicated that fucose-capture is a reliable, as well as rapid and convenient (<10 min operation on the whole procedure), approach for isolation of tumor-derived exosomes from serum, and the detection of fucosylated exosomal miRNAs might pave a prospective way for the early diagnosis of LUAD.

### Identification of differentially expressed miRNAs in serum-derived exosomes

To find the circulating exosomal miRNAs available for early LUAD diagnosis, small RNA sequencing was performed for serum exosomes isolated from eight HCs, eight BPNs, and eight early LUAD subjects. As a result, we identified 95 differentially expressed miRNAs (DEmiRs) in the exosomes of the early LUAD group compared to HC group and 72 DEmiRs in the exosomes of the early LUAD group compared to BPN group (*P* < 0.05). Among them, there were 38 overlapping DEmiRs in the early LUAD group compared with normal control (NC) group (NC: BPNs and HCs), including 10 upregulated DEmiRs and 28 downregulated DEmiRs (**Table 2**). Hierarchical clustering heatmap of these DEmiRs indicated that all early LUAD samples were correctly classified (**Figure S2**). The principal component analysis (PCA) of overlapping DEmiRs can well distinguish the LUAD group from these three groups (**Figure S3**).

**Table 2:** Overlapping differentially expressed miRNAs (DEmiRs) between DEmiRs (*P* < 0.05, LUAD *vs*. BPN) and DEmiRs (*P* < 0.05, LUAD *vs*. HC).

miRNA ID	LUAD vs. BPN		LUAD vs. HC		miRNA ID	LUAD vs. BPN		LUAD vs. HC	
	Fold change	*P*-value	Fold change	*P*-value		Fold change	*P*-value	Fold change	*P*-value
hsa-miR-4732-5p	2.22	0.003	2.183	0.002	hsa-miR-378i	0.554	0.013	0.477	0.004
hsa-miR-92b-3p	1.775	0.016	1.812	0.001	hsa-miR-27b-3p	0.55	0.015	0.334	0.006
hsa-miR-451a	1.732	0.005	2.822	0	hsa-miR-625-3p	0.538	0.007	0.471	0
hsa-miR-1180-3p	1.603	0.014	1.476	0.039	hsa-miR-378c	0.531	0.016	0.382	0.012
hsa-let-7i-5p	1.539	0.001	1.569	0.01	hsa-miR-27a-3p	0.511	0.008	0.569	0.042
hsa-miR-4732-3p	1.52	0.048	2.417	0.001	hsa-miR-143-3p	0.504	0.008	0.461	0.036
hsa-miR-16-2-3p	1.452	0.009	1.93	0.002	hsa-miR-191-3p	0.479	0.004	0.547	0.005
hsa-miR-16-5p	1.427	0.048	1.601	0.032	hsa-miR-378f	0.478	0.05	0.343	0.008
hsa-miR-486-5p	1.373	0.047	1.796	0	hsa-miR-30a-5p	0.475	0.012	0.464	0.033
hsa-miR-92a-3p	1.364	0.001	1.345	0.021	hsa-miR-23b-3p	0.466	0.016	0.369	0.006
hsa-miR-340-5p	0.715	0.007	0.514	0.007	hsa-miR-139-5p	0.462	0.002	0.484	0.016
hsa-miR-361-3p	0.709	0.046	0.717	0.032	hsa-miR-760	0.458	0	0.41	0.006
hsa-miR-423-3p	0.678	0.013	0.597	0.006	hsa-miR-9985	0.445	0.012	0.261	0.001
hsa-miR-23a-3p	0.649	0.018	0.503	0.01	hsa-miR-99a-5p	0.43	0.02	0.337	0.026
hsa-miR-378a-3p	0.616	0.016	0.421	0	hsa-miR-139-3p	0.424	0.026	0.248	0.005
hsa-let-7d-3p	0.613	0.006	0.617	0.015	hsa-miR-378e	0.395	0.045	0.217	0.016
hsa-miR-24-3p	0.601	0.008	0.536	0.001	hsa-miR-125b-2-3p	0.389	0.034	0.206	0.011
hsa-miR-6131	0.59	0.039	0.259	0.002	hsa-miR-23b-5p	0.241	0.003	0.188	0.007
hsa-miR-1307-3p	0.563	0.005	0.418	0	hsa-miR-30c-1-3p	0.109	0.04	0.061	0.008

### Candidate exosomal miRNA selection through the screening cohort

**Figure 2:**
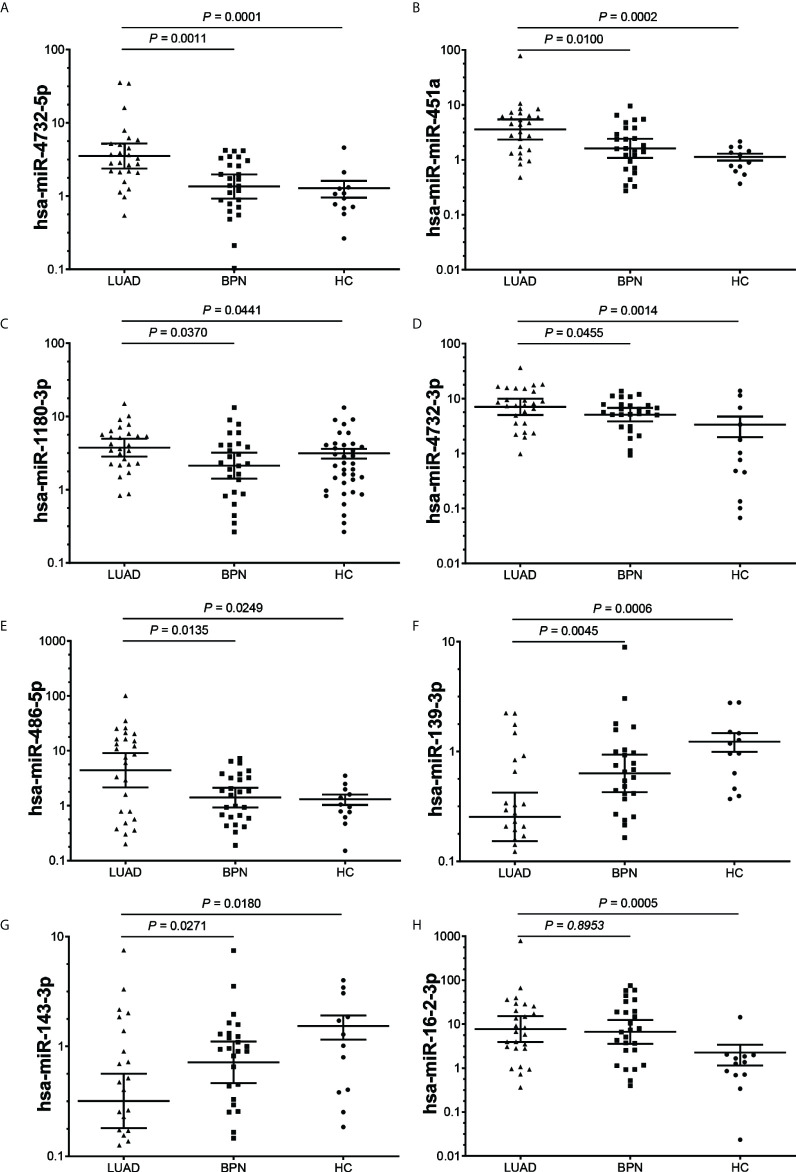
The expression levels of serum exosomal DEmiRs in the early LUAD, BPN and HC groups. **(A–G)**, The expression levels of 7 exosomal DEmiRs in LUAD group were significantly different from those in the BPN and HC groups. **(H)**, The DEmiRs was significantly up-regulated in LUAD group compared to that of HC group, whereas it did not show significant difference in LUAD group compared to BPN group. DEmiR, differentially expressed miRNA; LUAD, lung adenocarcinoma; BPN, benign pulmonary nodule; HC, healthy control.

Twelve exosomal DEmiRs were further screened by qRT-PCR in a screening cohort comprising 26 early LUAD samples and 38 NC samples (12 HCs and 26 BPNs). We selected a stable high-abundance miRNA (hsa-miR-20a) as an endogenous reference because its expression was highly consistent in all samples. The expression levels of seven exosomal DEmiRs (hsa-miR-4732-5p, hsa-miR-451a, hsa-miR-1180-3p, hsa-miR-4732-3p, hsa-miR-486-5p, hsa-miR-139-3p, and hsa-miR-143-3p) in the early LUAD group were significantly different from those in the BPN and HC groups (**Figures 2A–G**). These qRT-PCR results were consistent with the sequencing results. Furthermore, exosomal hsa-miR-16-2-3p was significantly upregulated in the serum exosomes of the LUAD patients compared to that of HC donors, whereas it did not show a significant difference in LUAD patients compared to BPN controls (**Figure 2H**). However, hsa-let-7i-5p, hsa-miR-139-5p, hsa-miR-378i, and hsa-miR-760 did not show any significant differences (**Figure S4**).

The AUC was calculated to evaluate the diagnostic performance of the seven exosomal DEmiRs (**Table 3**), of which four exosomal DEmiRs (hsa-miR-4732-5p, hsa-miR-451a, hsa-miR-486-5p, and hsa-miR-139-3p) yielded an AUC >0.7 (both sensitivity and specificity >0.65). Furthermore, binary logistic regression was utilized to combine these miRNA candidate biomarkers, and the combined detection further improved the AUC to 0.9332, holding promise as potential candidate biomarkers for early LUAD.

**Table 3:** Diagnostic performances of the seven exosomal DEmiRs for early LUAD.

MiRNA ID	AUC	*P*-value	Cutoff	Sensitivity %	Specificity %
hsa-miR-4732-5p	0.7935	<0.0001	>2.034	80.77	71.05
hsa-miR-451a	0.7546	0.0006	>1.727	73.08	65.79
hsa-miR-1180-3p	0.6811	0.0145	>3.426	59.26	66.67
hsa-miR-4732-3p	0.6989	0.0072	>6.506	65.38	65.79
hsa-miR-486-5p	0.7075	0.0051	>2.020	65.38	68.42
hsa-miR-139-3p	0.7728	0.0002	<0.3547	73.08	86.05
hsa-miR-143-3p	0.6959	0.0075	<0.5830	66.67	65.79
four-miRNAs	0.9332	<0.0001	>0.2149	96.15	78.95

AUC, area under the curve; four miRNAs, hsa-miR-4732-5p + hsa-miR-451a + hsa-miR-486-5p + hsa-miR-139-3p.

### The potential diagnostic value of candidate miRNAs was analyzed by the validating cohort

**Figure 3:**
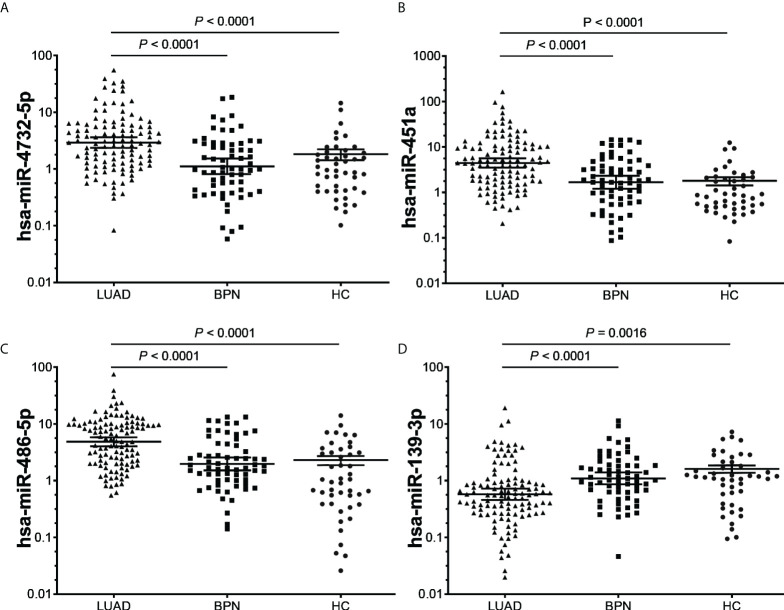
RT-qPCR validation of the four candidate miRNAs **(A)**, hsa-miR-4732-5p; **(B)**, hsa-miR-451a; **(C)**, hsa-miR-486-5p; **(D)**, hsa-miR-139-3p) in the validating cohort (n = 222). **(A–D)**, LUAD, lung adenocarcinoma; BPN, benign pulmonary nodule; HC, healthy control.

After analysis of the discovery and screening cohorts, four candidate miRNAs were selected for further verification. A validating cohort comprising 110 NC subjects (46 HCs and 64 BPNs) and 112 early LUAD patients were enrolled. RT-qPCR showed that exosomal hsa-miR-4732-5p, hsa-miR-451a, and hsa-miR-486-5p were significantly upregulated, whereas exosomal hsa-miR-139-3p was markedly downregulated in early LUAD patients compared with HC and BPN individuals (**Figure 3**).

**Figure 4:**
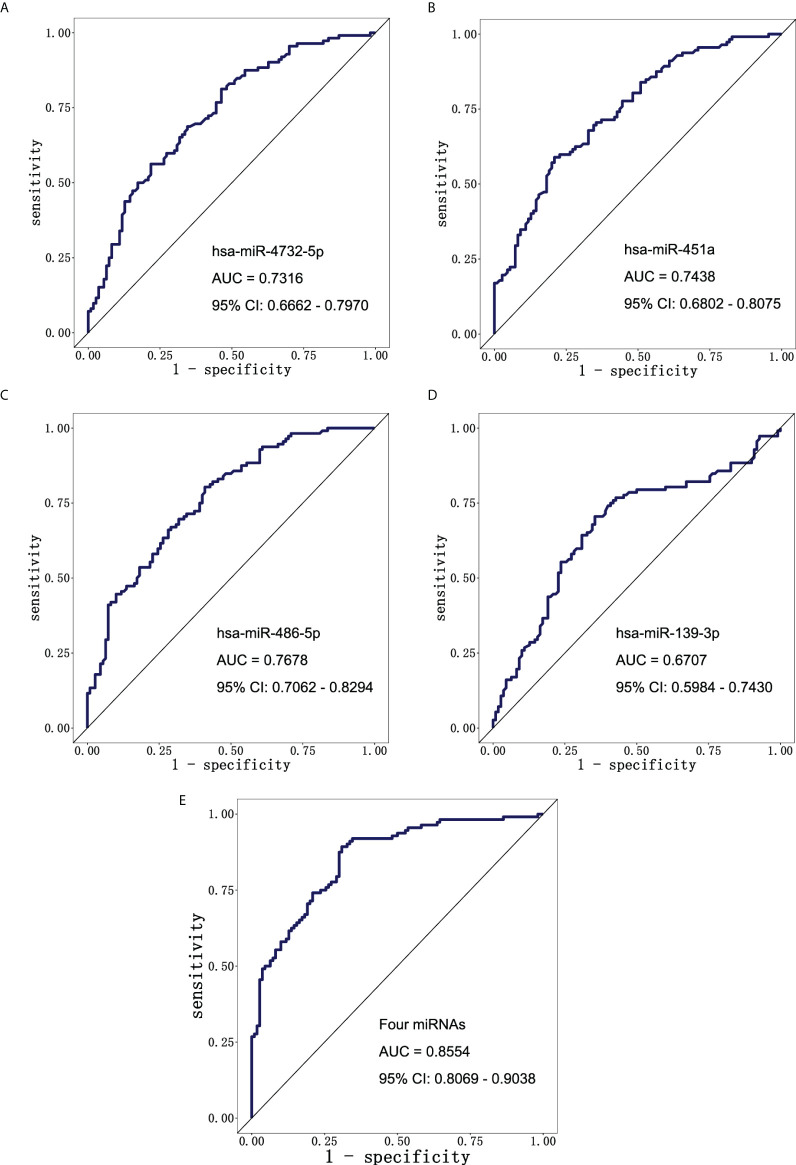
The diagnostic potential of candidate miRNAs. ROC curve analysis of **(A)** hsa-miR-4732-5p, **(B)** hsa-miR-451a, **(C)** hsa-miR-486-5p, and **(D)** hsa-miR-139-3p to differentiate LUAD patients (n = 112) and NCs (n = 110). **(E)** ROC curve analysis of combining four candidate miRNAs to differentiate LUAD patients (n = 112) from NCs (n = 110). ROC, receiver operating characteristic; LUAD, lung adenocarcinoma; NCs, normal controls.

To further explore the diagnostic value of miRNAs for early LUAD, ROC curve analysis was performed for the above four exosomal miRNA candidates. The AUC values of exosomal hsa-miR-4732-5p, hsa-miR-451a, hsa-miR-486-5p, and hsa-miR-139-3p were 0.7316 (95% CI: 0.6662–0.7970), 0.7438 (95% CI: 0.6802–0.8075), 0.7678 (95% CI: 0.7062–0.8294), and 0.6707 (95% CI: 0.5984–0.7430), respectively (**Figures 4A–D**). The result showed that the four-miRNA panel well distinguished the early LUAD group from the NC group with an AUC of 0.8554 (95% CI: 0.8069–0.9038) at a sensitivity of 91.07% (95% CI: 84.34%–95.08%) and a specificity of 66.36% (95% CI: 57.11%–74.51%) (**Figure 4E**). Additionally, the four-miRNA panel showed preferable performance than any of the miRNAs alone, presenting its potential as a non-invasive approach for early LUAD diagnosis.

### Pathway enrichment analysis of candidate miRNAs

**Figure 5:**
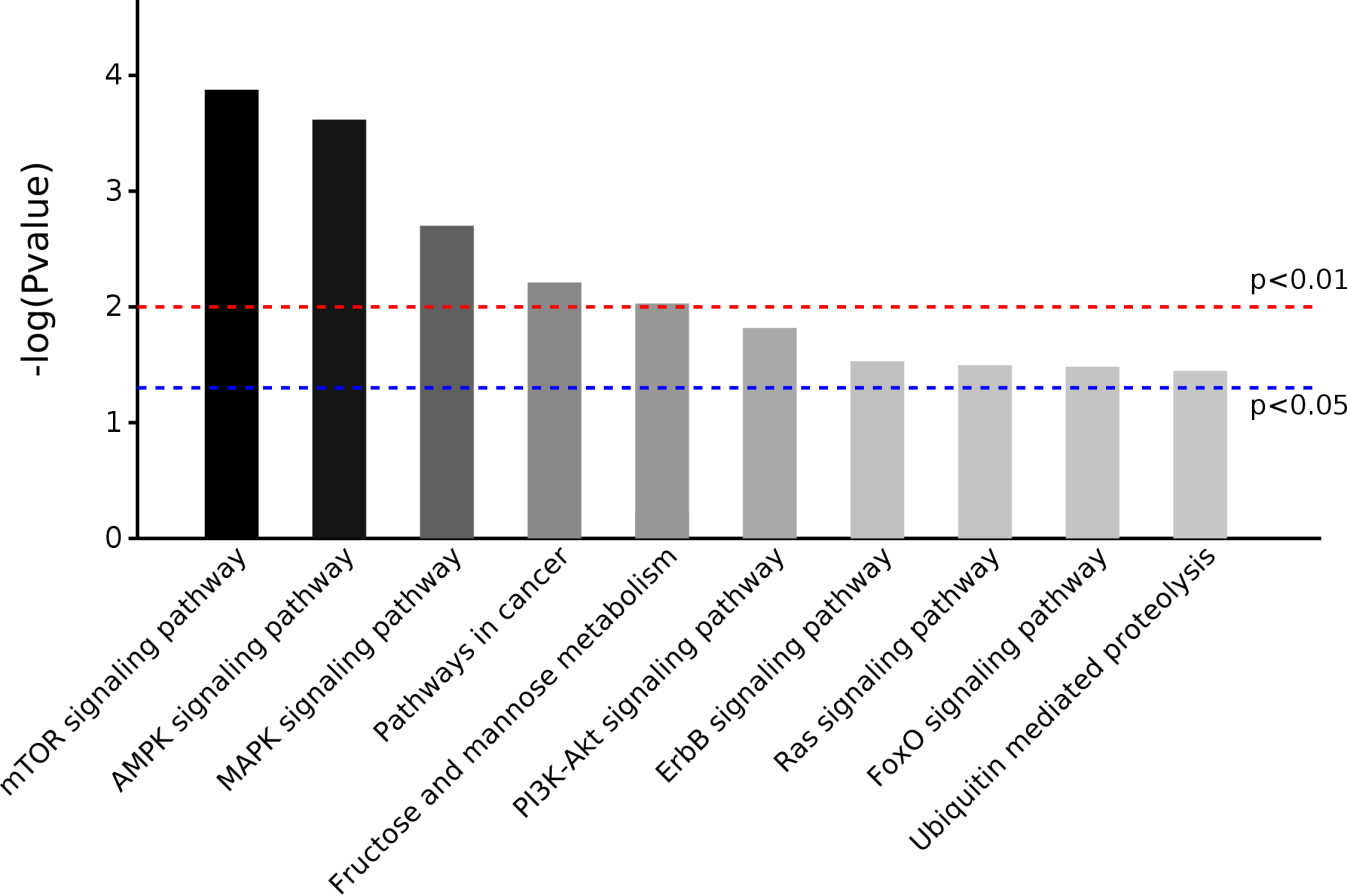
KEGG pathway analysis for predicted target genes of four candidate miRNAs.

To investigate the molecular function of four candidate miRNAs in LUAD, we performed KEGG pathway enrichment for target genes of these candidate miRNAs. KEGG pathway analysis results showed that 10 significant pathways (*P* < 0.05) were enriched, and most of them were cancer-related, such as mammalian target of rapamycin (mTOR) signaling pathway, Ras signaling pathway, and Pathways in cancer (**Figure 5**; **Table S3**).

**Figure 6:**
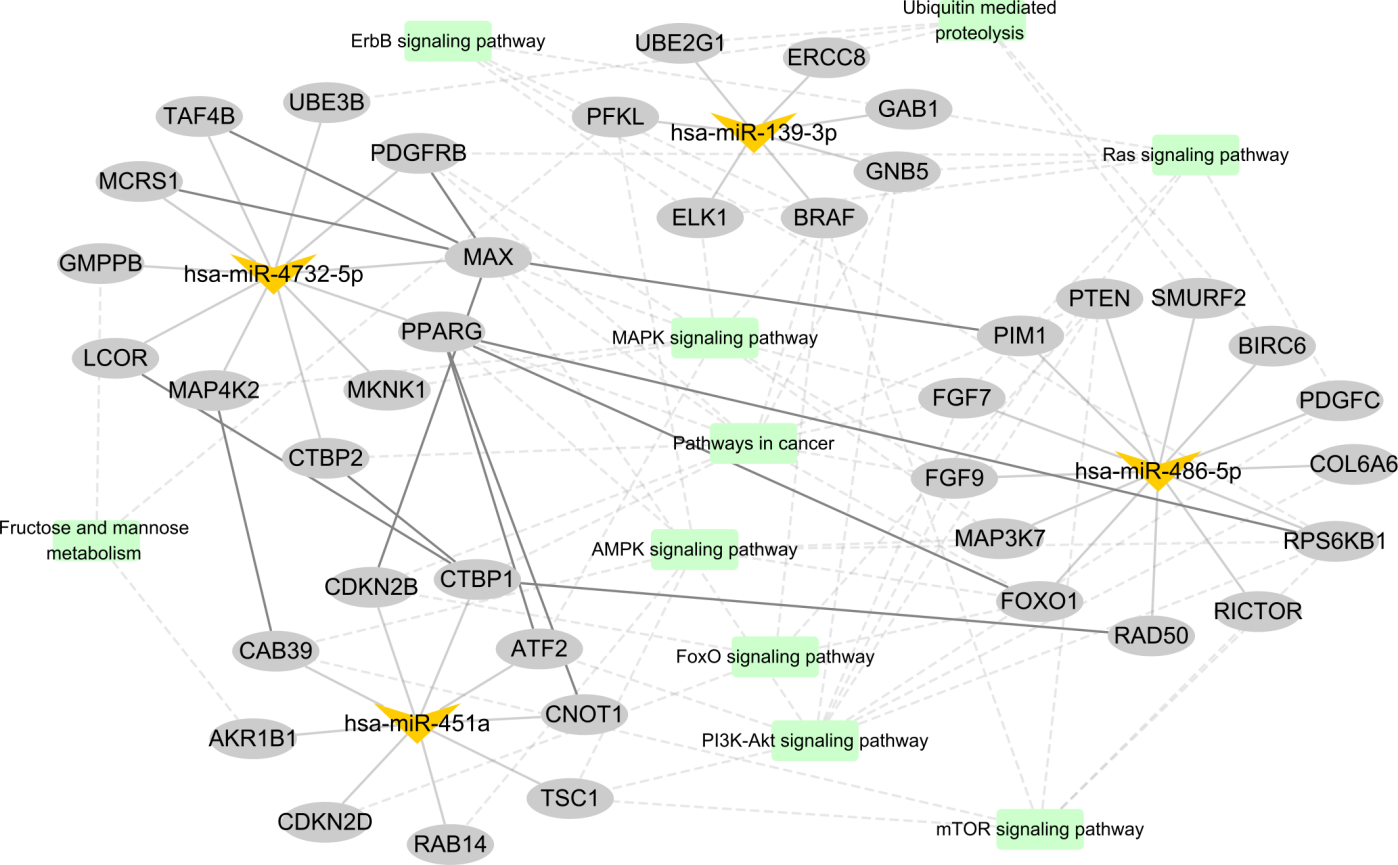
The candidate miRNA–mRNA interaction network. In this graphic, miRNAs, mRNAs, and KEGG pathways are represented with V shapes, ellipses, and rectangles, respectively. The thick solid lines represent protein–protein interactions from STRING database, the thin solid lines represent miRNA–target gene pairs, and the dotted lines represent the connected gene belonging to the KEGG pathway.

Furthermore, we constructed a miRNA–mRNA interaction network based on the regulatory relationship between miRNA and predicted target genes, protein–protein interaction in STRING database, and significantly enriched KEGG signaling pathway (**Figure 6**). Interestingly, *PPARG*, *CTBP1*, and *MAX* are the hub genes in this network, which have been confirmed by several studies to be involved in the occurrence and progress of malignancy. These results demonstrated that these four miRNAs not only served as diagnostic biomarkers but also might be involved in the tumorigenesis and development of LUAD.

## Discussion

Serum exosomal miRNAs have demonstrated their potential as non-invasive biomarkers for the diagnosis of lung cancer (19, 28, 29). However, despite advances in serum exosomal miRNAs for LUAD, few studies are carried out to define the performance of serum exosomal miRNAs in distinguishing early LUAD patients with BPNs more than HCs based on the miRNA profiling of serum exosomes by high-throughput sequencing up to now. Additionally, to the best of our knowledge, the absence of an effective approach for enriching tumor-derived exosomes from serum also strictly limits the application perspective of serum exosomal miRNAs in the early diagnosis of LUAD patients.

As a result, we first intend to solve the challenge of effectively enriching tumor-derived exosomes from serum in this study. It has been well demonstrated that some exosomes can gain access to the blood circulation from early primary tumor cells. These tumor-derived exosomes contain specific cargoes from tumor cells, which may provide valuable indicators involved in the tumorigenesis and development of malignancies (10). Therefore, how to effectively capture and enrich tumor-derived exosomes from complex blood systems is of great significance for the improvement of early diagnostic efficiency for patients with malignancies. Traditional isolation methods of exosomes are mainly based on exosome size and buoyancy density, including UC, microfiltration, and size exclusion chromatography, to name a few (30–32). Unfortunately, these isolation methods based on physical properties cannot well distinguish tumor-derived exosomes from benign exosomes. Recently, the immunoaffinity enrichment methods based on a highly specific interaction with exposed components on the surface of exosomes are emerging as the most promising approach for the capture of tumor-derived exosomes (33, 34). It is well known that aberrantly high expression of fucosyl transferase existed in most malignancies, which markedly elevated fucosylation of membrane surface proteins of extracellular vesicles derived from tumor cells (35). Therefore, developing the immunoaffinity enrichment method to capture the elevated fucosylation component on the surface of exosomes might provide an ideal approach for enriching the tumor-derived exosomes from sera of patients with LUAD. Encouragingly, in this study, we successfully captured and isolated tumor-derived exosomes from serum by coupling fucose-specific lentil lectin (LCA) to magnetic beads. The results showed that immunoaffinity fucose capture is a reliable, as well as a rapid and convenient, approach for the isolation of tumor-derived exosomes from serum, suggesting that the detection of serum fucosylated exosomal miRNAs might pave a new prospective way for the early diagnosis of LUAD.

Especially mentioned, recent studies have strongly emphasized the role of serum exosomal miRNAs as diagnostic biomarkers for early LUAD. For example, by investigating the serum biomarkers of 23 LUAD patients and 16 healthy subjects, Feng et al. (36) successfully identified several serum exosomal miRNAs as the emerging biomarkers for early diagnosis of LUAD, including miR-21-5p (AUC = 0.97, 95% CI: 0.846–0.99), miR-126-3p (AUC = 0.91, 95% CI: 0.77–0.98), and miR-140-5p (AUC = 0.88, 95% CI: 0.73–0.97). Han et al. (37) demonstrated the potential of the combination of serum exosomal miR-342-5p and miR-574-5p for distinguishing LUAD patients at an early stage from HCs, with an AUC up to 0.813 (95% CI: 0.7249–0.9009). Additionally, a panel of four serum exosomal miRNAs (miR-133a-3p, miR-584-5p, miR-10b-5p, and miR-221-3p) has been reported to present AUC values of 0.734, 0.803, and 0.894 for LUAD patients compared to healthy volunteers in the training, testing, and verification cohorts, respectively (38). Nevertheless, these above studies meet the limitations of a relatively small sample size and the oversight of BPN controls. In this current prospective study, we performed small RNA sequencing to obtain some candidate miRNAs for early LUAD. Then, by further qPCR verification in screening and validating cohorts, we successfully established a miRNA panel composed of four serum fucosylated exosomal miRNAs (hsa-miR-4732-5p, hsa-miR-451a, hsa-miR-486-5p, and hsa-miR-139-3p) as a promising diagnostic approach for early LUAD, with an AUC as high as 0.8554 in well distinguishing early LUAD patients from BPNs more than HCs at a sensitivity of 91.07% and a specificity of 66.36%. Interestingly, our previous study also showed that the expression level of serum exosomal hsa-mir-486-5p in early NSCLC was significantly upregulated, which presents good diagnostic value for patients with early NSCLC (39).

Moreover, it was reported that the *EVI5* oncogene was directly regulated by hsa-mir-486-5p, and hsa-mir-486-5p–*EVI5* axis affected the NSCLC migration and invasion through the *TGF-β*/*Smad* signaling pathway (40). hsa-miR-451a has also been shown to have an association with lung cancer. Yao et al. (41) demonstrated that hsa-miR-451a derived from extracellular vesicles was significantly increased in plasma of LUAD patients, compared to HCs, and was available for LUAD diagnosis. Especially mentioned, hsa-miR-451a was reported to play a key role in inhibiting the migration and invasion of NSCLC cells by regulating *ATF2* expression (42). However, the effects of hsa-miR-4732-5p and hsa-miR-139-3p on lung cancer have not yet been reported up to now and is worth exploring in our future study.

Acknowledgedly, there are several potential limitations existing in this study. Firstly, due to the relatively small number of serum samples, our results need to be further demonstrated in a larger sample cohort and in a multicenter study. Secondly, due to the absence of follow-up information, the clinical value of candidate miRNAs for prognosis evaluation of LUAD patients was not indicated. Finally, effects on LUAD cells should be clearly clarified so as to better understand the role of these candidate miRNAs on LUAD development.

In conclusion, we first isolated and enriched tumor-derived exosomes from serum by a developed fucose-captured technique, which contributes to obtaining valuable exosomal biomarkers with higher sensitivity and/or specificity. Furthermore, we successfully developed a four-miRNA panel of fucosylated exosomes for early LUAD diagnosis. These findings will contribute to the improvement of efficiency of serological screening for early LUAD, thereby reducing patient mortality.

## Data availability statement

The data presented in the study are deposited in the NCBI repository, SRA accession number PRJNA847004.

## Ethics statement

This study was conducted in accordance with the International Ethical Guidelines for Biomedical Research Involving Human Subjects (CIOMS) and approved by the Institutional Review Board (IRB) of Fujian Province hospital (ID: K2018-12-040). The patients/participants provided their written informed consent to participate in this study.

## Author contributions

XC: Software, Validation, Visualization, Methodology, Writing–original draft, Project administration. LY: Formal analysis, Investigation, Methodology, Writing–original draft. KH: Formal analysis, Investigation, Methodology, Writing–original draft. XY: Formal analysis, Investigation, methodology. MT: Formal analysis, Investigation, Methodology. LC: Formal analysis, Investigation, Methodology. LZ: Formal analysis, Investigation, Methodology. XP: Resources, Data curation. QG: Conceptualization, Resources, Formal analysis, Supervision, Validation, Methodology. YH: Conceptualization, Resources, Formal analysis, Supervision, Funding acquisition, Validation, Writing–original draft, Project administration. All authors contributed to the article and approved the submitted version.

## Funding

This work was supported by Medical Vertical Project of Fujian Province (Grant No. 2020CXB001), High-level Hospital Foster Grant of Fujian Provincial Hospital (Grant No. 2020HSJJ06), Key project of natural science foundation of Fujian province (Grant No. 2022J02048).

## Conflict of interest

Author KH and QG were employed by Beijing Glyexo Gene Technology Co.,Ltd

The remaining authors declare that the research was conducted in the absence of any commercial or financial relationships that could be construed as a potential conflict of interest.

## Publisher’s note

All claims expressed in this article are solely those of the authors and do not necessarily represent those of their affiliated organizations, or those of the publisher, the editors and the reviewers. Any product that may be evaluated in this article, or claim that may be made by its manufacturer, is not guaranteed or endorsed by the publisher.
