# Supplementary material for: Fucosylated exosomal miRNAs as promising biomarkers for the diagnosis of early lung adenocarcinoma
Source: Front Oncol. 2022 Aug 12;12:935184. doi: 10.3389/fonc.2022.935184 (PMC9414872; doi:10.3389/fonc.2022.935184)
Supplement: Supplementary file 1 [file DataSheet_1.docx]

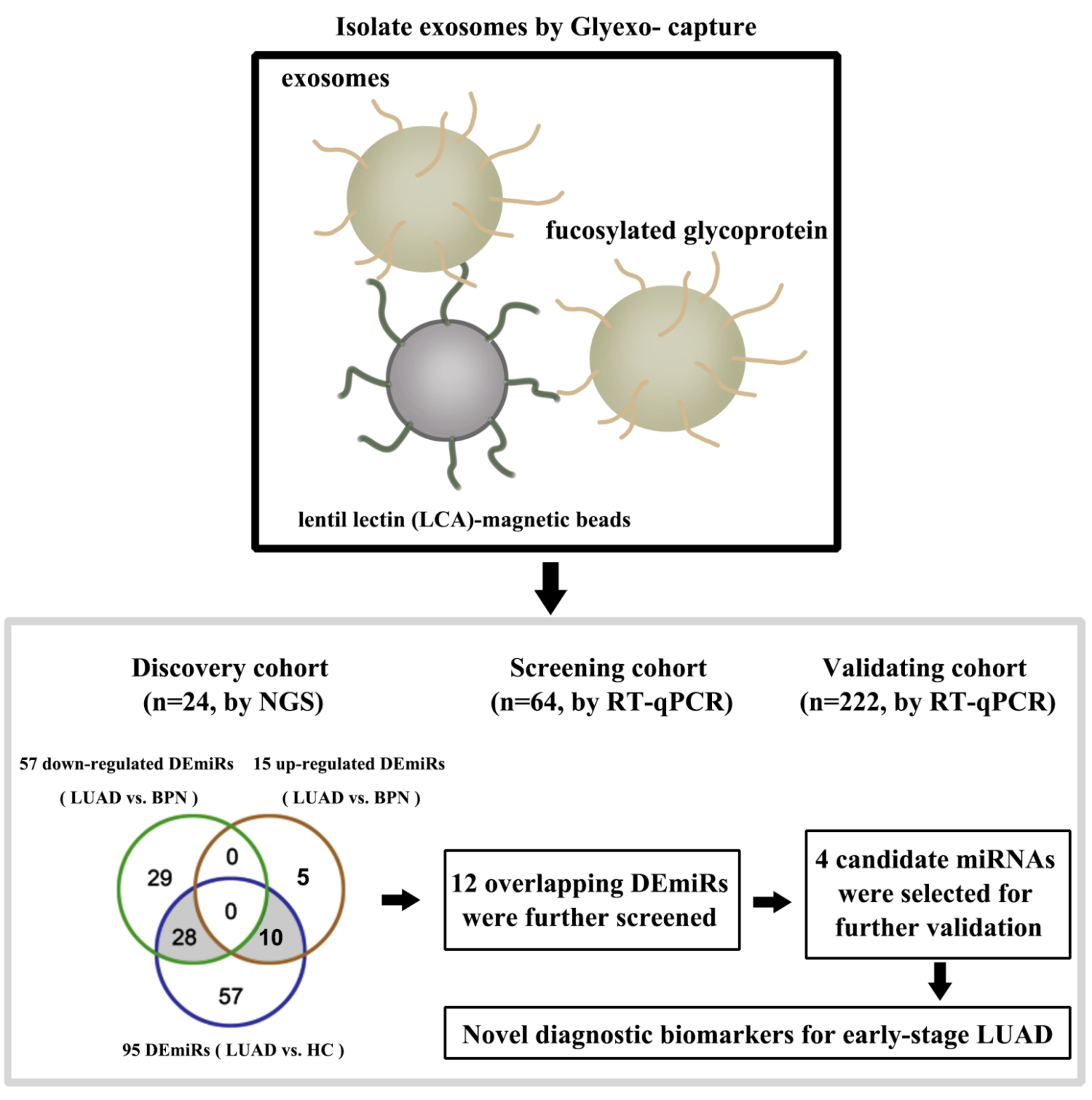


Figure S1. The screening workflow for identifying exosomal miRNAs for diagnosing early LUAD. Abbreviations: LUAD, lung adenocarcinoma; BPN, benign pulmonary nodule; HC, healthy control.

**

**

Figure S2. Heatmap of exosomal Overlapping DEmiRs between DEmiRs (p<0.05, LUAD vs. BPN) and DEmiRs (p<0.05, LUAD vs. HC). Columns represent samples from different groups. Rows stand for the 38 differential miRNAs. The color of each entry is determined by the miRNA expression levels, ranging from blue (negative values) to orange (positive values). Abbreviations: DEmiRs, differentially expressed miRNAs; LUAD, lung adenocarcinoma; BPN, benign pulmonary nodule; HC, healthy control.

**
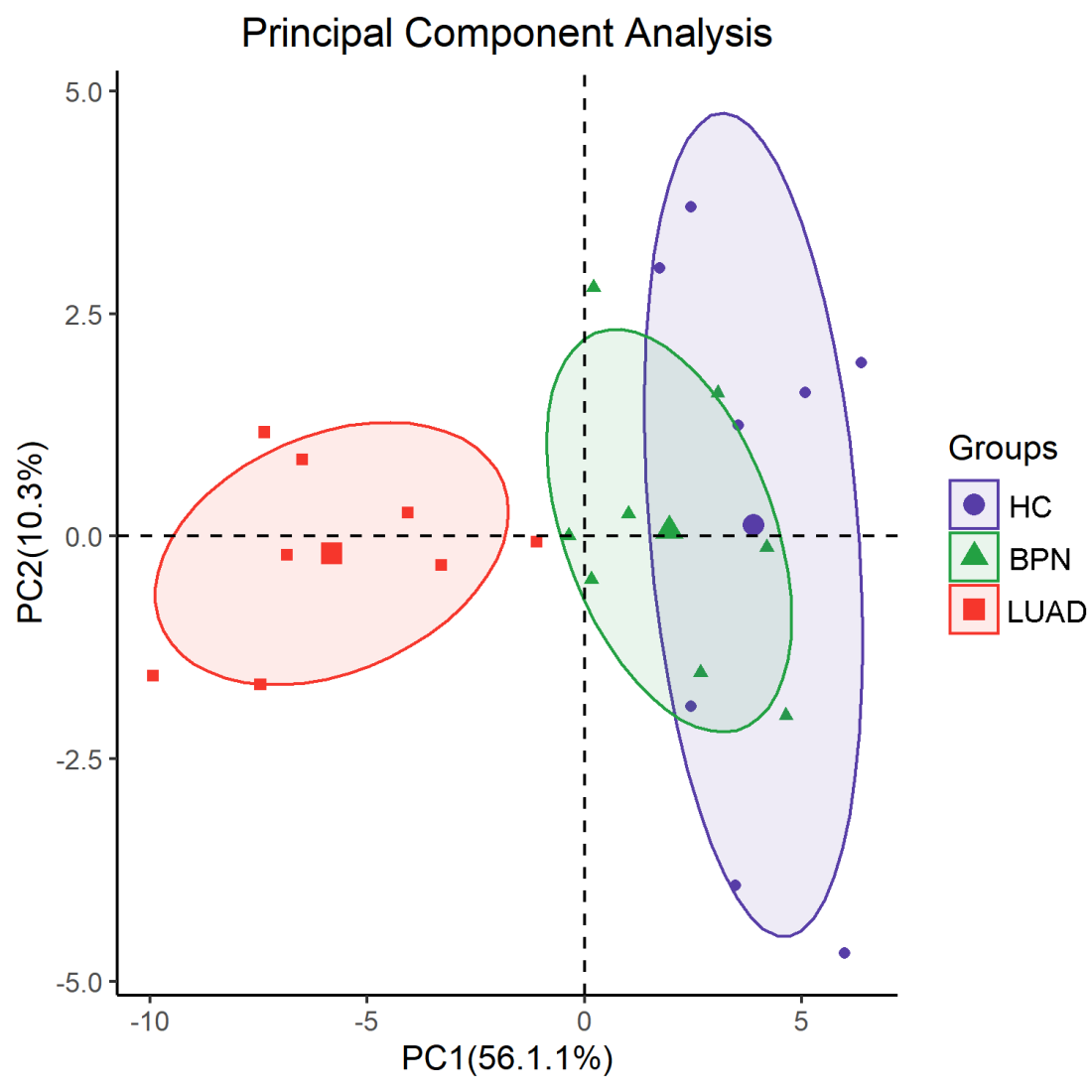
**

Figure S3. Principal component analysis (PCA) including the 38 overlapping DEmiRs. Abbreviations: DEmiRs, differentially expressed miRNAs; LUAD, lung adenocarcinoma; BPN, benign pulmonary nodule; HC, healthy control.


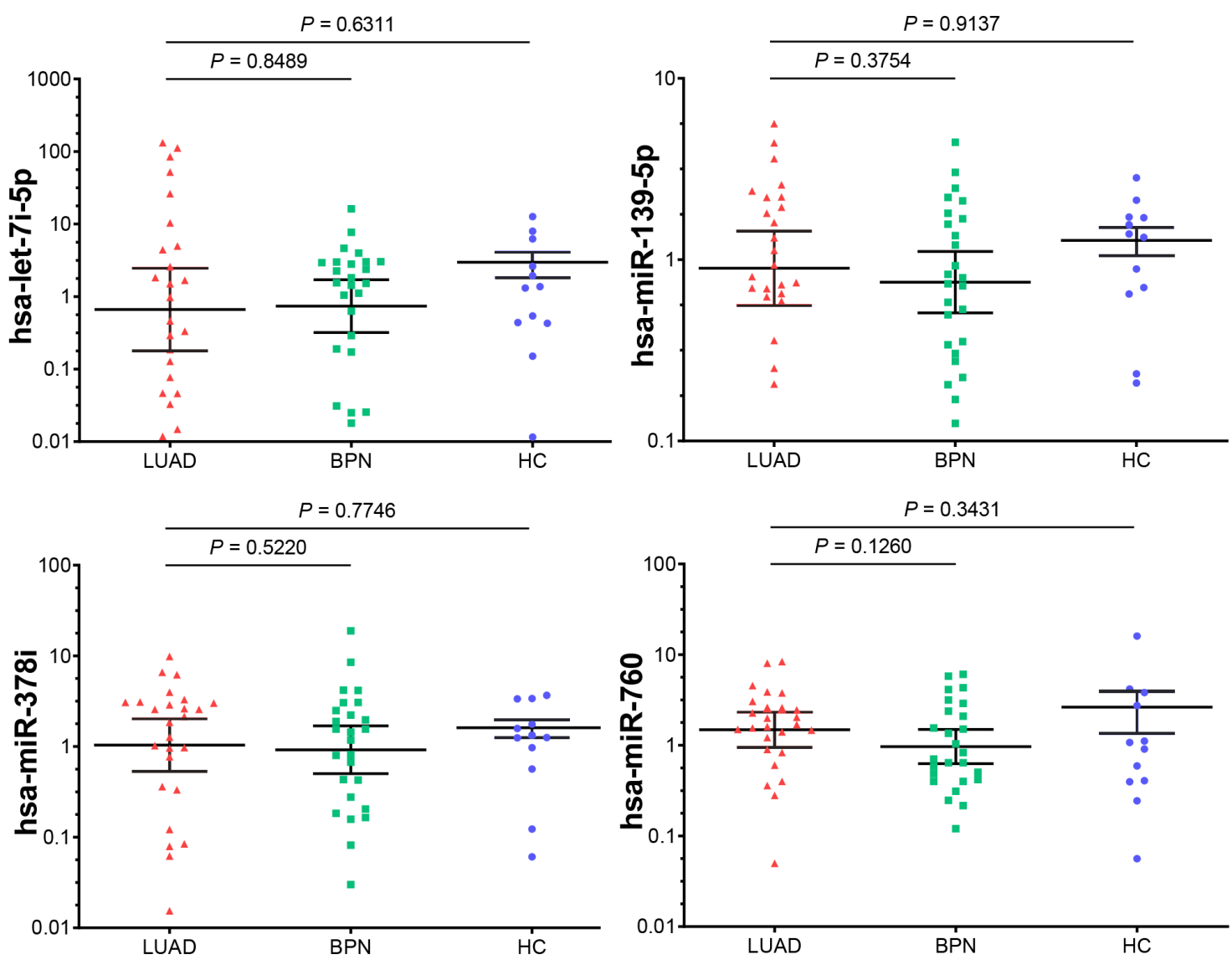


Figure S4. Four exosomal DEmiRs showed no significant difference in the early LUAD, BPN and HC groups. Abbreviations: LUAD, lung adenocarcinoma; BPN, benign pulmonary nodule; HC, healthy control.
