# Supplementary material for: Fucosylated exosomal miRNAs as promising biomarkers for the diagnosis of early lung adenocarcinoma
Source: Front Oncol. 2022 Aug 12;12:935184. doi: 10.3389/fonc.2022.935184 (PMC9414872; doi:10.3389/fonc.2022.935184)
Supplement: Supplementary file 2 [file Table_1.docx]

Table S1. The program for isolating Fucosylated exosomes

| Step | Well | Solution | Volume  (µL) | Mixing speed | Mixing time  (minutes) | Precipitation time  (seconds) |
| --- | --- | --- | --- | --- | --- | --- |
| MIX | 1 | MBL | 750 | low | 1 | 240 |
| WASH | 2 | WBL | 600 | low | 1 | 1 |
| ELUTE | 3 | EBL | 250 | low | 1 | 240 |

Abbreviations: MBL, LCA coupled magnetic beads solution; WBL, washing solution; EBL, elution buffer.

Table S2. List of primers used in this study.

| miRNA | primer sequence |
| --- | --- |
| hsa-miR-4732-5p | CTGTAGAGCAGGGAGCAGGAAG |
| hsa-miR-451a | CCGCGAAACCGTTACCATTACTGAGTT |
| hsa-miR-1180-3p | TTCCGGCTCGCGTGGGTGTGT |
| hsa-miR-4732-3p | GCCCTGACCTGTCCTGTTCTG |
| hsa-miR-486-5p | CGTCCTGTACTGAGCTGCCC |
| hsa-miR-16-2-3p | CGCGCCAATATTACTGTGCTGCTTTA |
| hsa-let-7i-5p | CCGCTGAGGTAGTAGTTTGTGCTGTT |
| hsa-miR-378i | CCGACTGGACTAGGAGTCAGAAGG |
| hsa-miR-143-3p | CCGTGAGATGAAGCACTGTAGCTC |
| hsa-miR-139-5p | TCTACAGTGCACGTGTCTCCAGT |
| hsa-miR-760 | AATCGGCTCTGGGTCTGTGG |
| hsa-miR-139-3p | AATATATTGGAGACGCGGCCCTGTT |
| hsa-miR-20a | CCGCGTAAAGTGCTTATAGTGCAGGTAG |

Table S3. List of KEGG pathway enrichment for target genes of 4 diagnostic miRNAs.

| Pathway Name | Pathway ID | Pvalue | Genes |
| --- | --- | --- | --- |
| mTOR signaling pathway | hsa04150 | 1.33E-04 | PTEN\|1;TSC1\|1;RPS6KB1\|1;RICTOR\|1;BRAF\|1;CAB39\|1 |
| AMPK signaling pathway | hsa04152 | 2.41E-04 | PPARG\|1;MAP3K7\|1;TSC1\|1;RPS6KB1\|1;RAB14\|1;PFKL\|1;FOXO1\|1;CAB39\|1 |
| MAPK signaling pathway | hsa04010 | 1.99E-03 | MAX\|1;MAP3K7\|1;ELK1\|1;PDGFRB\|1;MKNK1\|1;BRAF\|1;MAP4K2\|1;FGF9\|1;FGF7\|1;ATF2\|1 |
| Pathways in cancer | hsa05200 | 6.15E-03 | MAX\|1;PTEN\|1;CTBP1\|1;PPARG\|1;GNB5\|1;CDKN2B\|1;CTBP2\|1;PDGFRB\|1;BRAF\|1;FOXO1\|1;FGF9\|1;FGF7\|1 |
| Fructose and mannose metabolism | hsa00051 | 9.35E-03 | GMPPB\|1;PFKL\|1;AKR1B1\|1 |
| PI3K-Akt signaling pathway | hsa04151 | 1.52E-02 | PTEN\|1;GNB5\|1;TSC1\|1;PDGFRB\|1;RPS6KB1\|1;COL6A6\|1;PDGFC\|1;FGF9\|1;FGF7\|1;ATF2\|1 |
| ErbB signaling pathway | hsa04012 | 2.95E-02 | ELK1\|1;RPS6KB1\|1;GAB1\|1;BRAF\|1 |
| Ras signaling pathway | hsa04014 | 3.19E-02 | GNB5\|1;ELK1\|1;PDGFRB\|1;GAB1\|1;PDGFC\|1;FGF9\|1;FGF7\|1 |
| FoxO signaling pathway | hsa04068 | 3.28E-02 | PTEN\|1;CDKN2B\|1;BRAF\|1;FOXO1\|1;CDKN2D\|1 |
| Ubiquitin mediated proteolysis | hsa04120 | 3.56E-02 | SMURF2\|1;ERCC8\|1;BIRC6\|1;UBE3B\|1;UBE2G1\|1 |
